# The association between green space and cause-specific mortality in urban New Zealand: an ecological analysis of green space utility

**DOI:** 10.1186/1471-2458-10-240

**Published:** 2010-05-11

**Authors:** Elizabeth Richardson, Jamie Pearce, Richard Mitchell, Peter Day, Simon Kingham

**Affiliations:** 1 School of GeoSciences, The University of Edinburgh, Edinburgh, UK; 2 Section of Public Health and Health Policy, Faculty of Medicine, University of Glasgow, Glasgow, UK; 3 GeoHealth Laboratory, Department of Geography, University of Canterbury, Christchurch, New Zealand

## Abstract

**Background:**

There is mounting international evidence that exposure to green environments is associated with health benefits, including lower mortality rates. Consequently, it has been suggested that the uneven distribution of such environments may contribute to health inequalities. Possible causative mechanisms behind the green space and health relationship include the provision of physical activity opportunities, facilitation of social contact and the restorative effects of nature. In the New Zealand context we investigated whether there was a socioeconomic gradient in green space exposure and whether green space exposure was associated with cause-specific mortality (cardiovascular disease and lung cancer). We subsequently asked what is the mechanism(s) by which green space availability may influence mortality outcomes, by contrasting health associations for different types of green space.

**Methods:**

This was an observational study on a population of 1,546,405 living in 1009 small urban areas in New Zealand. A neighbourhood-level classification was developed to distinguish between usable (i.e., visitable) and non-usable green space (i.e., visible but not visitable) in the urban areas. Negative binomial regression models were fitted to examine the association between quartiles of area-level green space availability and risk of mortality from cardiovascular disease (*n *= 9,484; 1996 - 2005) and from lung cancer (*n *= 2,603; 1996 - 2005), after control for age, sex, socio-economic deprivation, smoking, air pollution and population density.

**Results:**

Deprived neighbourhoods were relatively disadvantaged in total green space availability (11% less total green space for a one standard deviation increase in NZDep2001 deprivation score, *p *< 0.001), but had marginally more usable green space (2% more for a one standard deviation increase in deprivation score, *p *= 0.002). No significant associations between usable or total green space and mortality were observed after adjustment for confounders.

**Conclusion:**

Contrary to expectations we found no evidence that green space influenced cardiovascular disease mortality in New Zealand, suggesting that green space and health relationships may vary according to national, societal or environmental context. Hence we were unable to infer the mechanism in the relationship. Our inability to adjust for individual-level factors with a significant influence on cardiovascular disease and lung cancer mortality risk (e.g., diet and alcohol consumption) will have limited the ability of the analyses to detect green space effects, if present. Additionally, green space variation may have lesser relevance for health in New Zealand because green space is generally more abundant and there is less social and spatial variation in its availability than found in other contexts.

## Background

Whilst individual characteristics are undoubtedly an important determinant of population health in an area, research has found that the residential environment has a significant independent influence on health outcomes [1]. A potentially important contextual factor that has recently attracted interest is that of access to natural environments, or 'green space' [2]. Green environments are associated with better self-perceived health [3-6], lower blood pressure [7], lower levels of overweight and obesity [8], lower levels of physician-assessed morbidity [9], as well as lower mortality risks [10]. Evidence for these associations has been found in a number of countries: the Netherlands [3,4], England [5], Australia [6], the USA [7], Scotland [8], and Japan [11]. In New Zealand no association was found between access to parks and individual-level BMI or physical activity levels [12] although the relationship has not been investigated for other types of green space or health outcomes.

Three key mechanisms have been proposed to explain how green space might influence health [2]. First, green space provides opportunities for physical activity (PA) [13,14], and increased PA levels are associated with reduced risks of physical and mental illnesses [15-17]. For instance, enhanced physical activity explained the association between green space and physical health in Adelaide, Australia [6]. Second, green space may benefit health by facilitating social contacts, for example through providing opportunities to meet others or participate in group activities [2,18]. Maas et al. [18] found that a lack of social contact partly mediated the association between low green space neighbourhoods and poor health in the Netherlands. If physical activity promotion or facilitation of social contact are key mechanisms in the relationship we would expect health to be more strongly related to the availability of green space that is usable (e.g., parks) than to all green space in general.

Third, exposure to green space can promote recovery from attention fatigue [19,20], and stress [21], and stress has been implicated in the aetiology of common chronic physical and mental illnesses [2,22]. These restorative benefits have been reported for subjects with only visual contact with green space [7,23], as well as those also having physical contact [7,24]. If these restorative psychosocial effects are the key mediators between green space and health we would expect health to be related to total green space availability, whether usable or not (e.g., agricultural land). Identifying whether health benefits are more strongly associated with usable or total green space will inform the causative mechanism debate and the development of public health policies and intervention strategies. Although creating a dichotomy between these potential mechanisms makes a useful framework for study it should be noted that they are not mutually exclusive. For instance, restorative and physical activity benefits may combine when exercising in green surroundings [24].

There is concern that locational access to health-promoting community resources, such as green space, is lower in socioeconomically deprived areas, and may be contributing to widening geographical inequalities in health [25]. There is some evidence that socioeconomically deprived communities have poorer green space availability than more affluent areas [26,27], which may partly explain the lower levels of physical activity in deprived communities [28]. In New Zealand, however, deprived communities in urban areas have better access to parks [29,30], but the socio-spatial patterning has not been investigated for usable green space in general, or total green space. Quantifying variations in usable and total green space exposure may therefore assist in understanding and addressing health inequalities.

We conducted a New Zealand-based study to contribute to the evidence base on the association between green space and health, and the underlying mechanisms that may bring about this relationship. Much of the existing evidence about green space and health has stemmed from European nations, with relatively similar social, economic and physical environments. We developed a novel and accurate neighbourhood-level measure of green space for urban areas of New Zealand, which differentiated between usable and non-usable types. The classification enabled us to address three research questions: (a) is there a socioeconomic gradient in green space exposure; (b) is there an association between green space availability and cause-specific mortality; and (c) what is the mechanism(s) by which green space availability may influence mortality outcomes?

We purposefully selected two causes of mortality with differing aetiologies: cardiovascular disease and lung cancer. Cardiovascular disease (CVD) is a leading cause of death in New Zealand, and has certain risk factors (inactivity and stress) which might be partly ameliorated by green space. Indeed, physical activity has been strongly associated with a reduced risk of CVD mortality in many studies [16]. Lung cancer (LC) is the most common cause of cancer mortality in New Zealand, but smoking is the main risk factor, and its relationship with physical activity is, at best, small [31]. We therefore hypothesised that CVD would be associated with green space whereas lung cancer mortality would not.

## Methods

Using a geographical information system (GIS) we developed a classification of green space for small areas across New Zealand that distinguished between usable and non-usable areas. We calculated the percentage coverage of these green space types for each urban neighbourhood and then investigated their patterning across the socioeconomic gradient and their relationships with cause-specific mortality, after adjusting for relevant confounders.

### Green space classification

**Figure 1:**
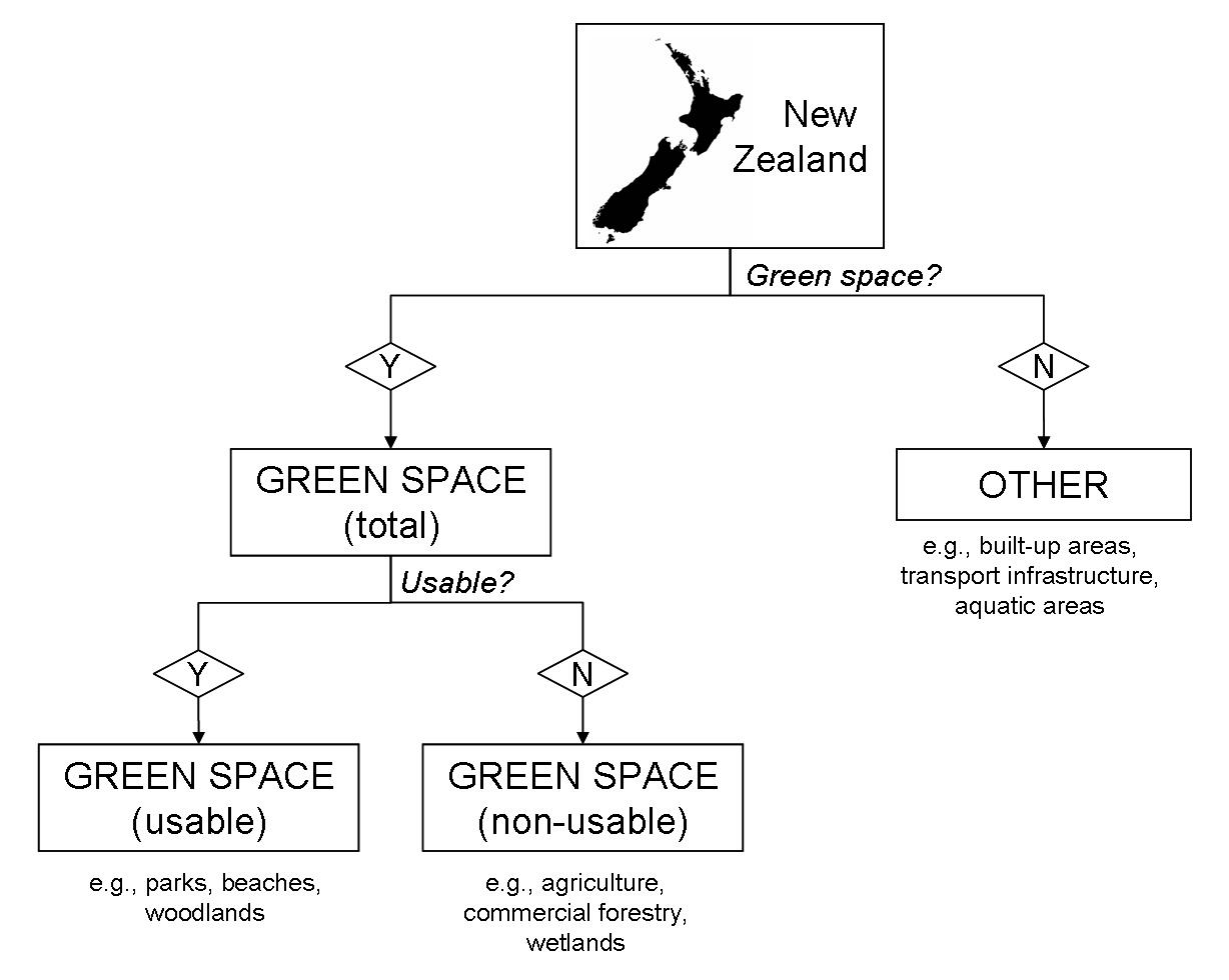
**Flowchart illustration of usable and non-usable green space classification system**.

Spatial land cover data sets for New Zealand were sought and processed using ArcMap GIS software (ESRI, Redlands, CA) to produce the green space classification. For the purposes of distinguishing usable and non-usable green space across the country we required data with both a good level of attribute information and national coverage. Three New Zealand-wide spatial data sets (with land areas represented as polygons) were obtained and integrated (Table 1). The Land Cover Data Base (LCDB2) data set gave contiguous national coverage but had the lowest resolution and provided the least attribute information; hence we augmented this data set with two more detailed but less contiguous data sets from the Department of Conservation (DOC) and Land Information New Zealand (LINZ). Our definition of green space included natural areas (e.g., parks, beaches, and fields) but excluded aquatic areas (e.g., lakes and the sea) as these are not generally treated as green space in the literature. The decision tree developed to produce our green space classification is shown in Figure 1.

**Table 1:** Data set specifications for green space classification.

Data set	Spatial resolution	Details
Department of Conservation (DOC) Conservation Boundaries data set (2003)	High	Legal boundaries of land administered by the DOC, and of land of interest to but not administered by the DOC. Attributes include legal designation (including specific Act) and site name.

Land Information New Zealand's (LINZ) Core Records System (2004)	High	Legal boundaries of land parcels across New Zealand, derived from the Core Records System's Survey, Title and Addresses data sets. Attributes include the purpose of any Statutory Actions on the parcels, although these purposes are not standardised, and are occasionally ambiguous.

Ministry for the Environment Land Cover Database 2 (LCDB2) (2001)	Lower resolution (intended scale 1: 50,000, minimum mapping unit = 1 ha).	61 land cover classes, derived from supervised and manual classification of Landsat 7 ETM+ satellite imagery and verified using some ground data. Specific land cover class provided as an attribute.

We began our classification process with the most informative data set: the DOC conservation area boundaries. Attribute information provided the legal status of each conservation area and permitted identification of usable green space (e.g., 'Scenic Reserve'), non-usable green space (e.g., 'Sanctuary Area') and other land (e.g., 'Administration Purpose').

The next most informative data set, the LINZ Core Records System, was then used to identify further green space areas from the remaining unclassified land. Attribute information for the 'purpose' of each LINZ parcel was used to identify usable and non-usable green space. Finally, the LCDB2 was used to identify any remaining unclassified areas. Usable green space was defined as 'urban parkland/open space', 'beaches', and any non-commercial forestry ('indigenous forest', 'deciduous hardwoods', or 'other exotic forest') that was either adjacent to other usable green space or was within 10 m of a road (i.e., accessible). Non-usable green space was defined as all other natural areas, including agricultural land, salt marsh, and commercial forestry.

Census Area Units (CAUs) were used as our small area geography for the analysis. CAUs are the second smallest census geography in New Zealand, and the smallest areal unit for which mortality data are disseminated. We restricted our analyses to urban areas because 71% of the New Zealand population lives in these areas (2.7 million people) [32]. We selected 1009 CAUs from the 2001 Census that were classified by Statistics New Zealand as being 'main urban areas' [32]. Using an intersect operation in ArcMap we then calculated the proportion of total and usable green space coverage within each CAU. These 1009 CAUs had a mean population in 2001 of 2630 and a mean area of 5 km^2^. As this area was equivalent to that of a circle with a radius of approximately 1.3 km our measure represented green spaces within relatively easy walking or cycling distance of CAU residents. Restricting our analyses to urban areas therefore had practical benefits for exposure classification, as green spaces within larger rural CAUs would be more widely dispersed, and would not all be within walking or cycling distance.

### Health data

We obtained anonymised, individual-level mortality data (including information on age, sex and domicile of residence at death) for every registered death between 1996 and 2005 from the New Zealand Ministry of Health. Individual deaths were matched to CAUs. Cardiovascular disease (CVD) and lung cancer (LC) mortality counts were generated by sex, age-group (15-44, 45-54, 55-64) and CAU. Analyses using more age-groups did not alter the results obtained. Denominator age-group and sex-specific population counts were extracted for each CAU from the 2001 census. The analysis was restricted to adults under 65 in order to study premature mortality. The total study population was 1,546,405 (in 2001), with 9,484 deaths from CVD and 2,603 from LC over the 10-year period.

### Confounders

In order to account for the strong influence of socioeconomic deprivation on the selected health outcomes we extracted area-level New Zealand Deprivation Index (NZDep2001) scores for the CAUs [33]. The NZDep2001 combines CAU-level census data on income, employment, communication, support, transport, qualifications, living space and home ownership [33]. The scores are scaled to have a mean of 1000 and a standard deviation of 100 index points. Smoking is an important risk factor for both CVD and LC, hence we adjusted for smoking by extracting counts of regular smokers from the 1996 and 2006 censuses and calculating an average percent smokers measure for each CAU.

We controlled for air pollution as a potential confounder, because greener places tend to be less polluted due to the reduced amount of land available to pollution-generating processes (e.g., traffic, domestic heating, and industry). We used a validated CAU-level measure of particulate matter with a median diameter less than 10 μm (PM_10_), the development of which is described elsewhere [34]. We also adjusted for population density (persons per hectare) as a measure of urbanity, because the green space and health relationship may vary with urbanity [3,5].

### Analysis

Due to over-dispersion (i.e., greater variance in the mortality data than expected), negative binomial regression models were used to model the relationship between CVD and LC mortality and the availability of different types of green space. The models were adjusted by age-group, sex, area-level socioeconomic status (NZDep2001), area-level smoking rate, area-level PM_10 _and population density. The age- and sex-specific population count in each CAU was entered as the exposure variable.

Incidence rate ratios (IRRs) and 95% confidence intervals (CIs) were calculated for quartile measures of green space availability (total and usable). The baseline model (model 1) adjusted for the confounding effects of age-group and sex in the relationship between green space availability quartiles and cause-specific mortality. Model 2 additionally controlled for area deprivation (NZDep2001 quintiles derived specifically for the subset of CAUs), model 3 for smoking rate (quartiles), model 4 for the air pollutant PM_10 _(quintiles), and model 5 for population density (quintiles).

## Results

### Green space classification

**Figure 2:**
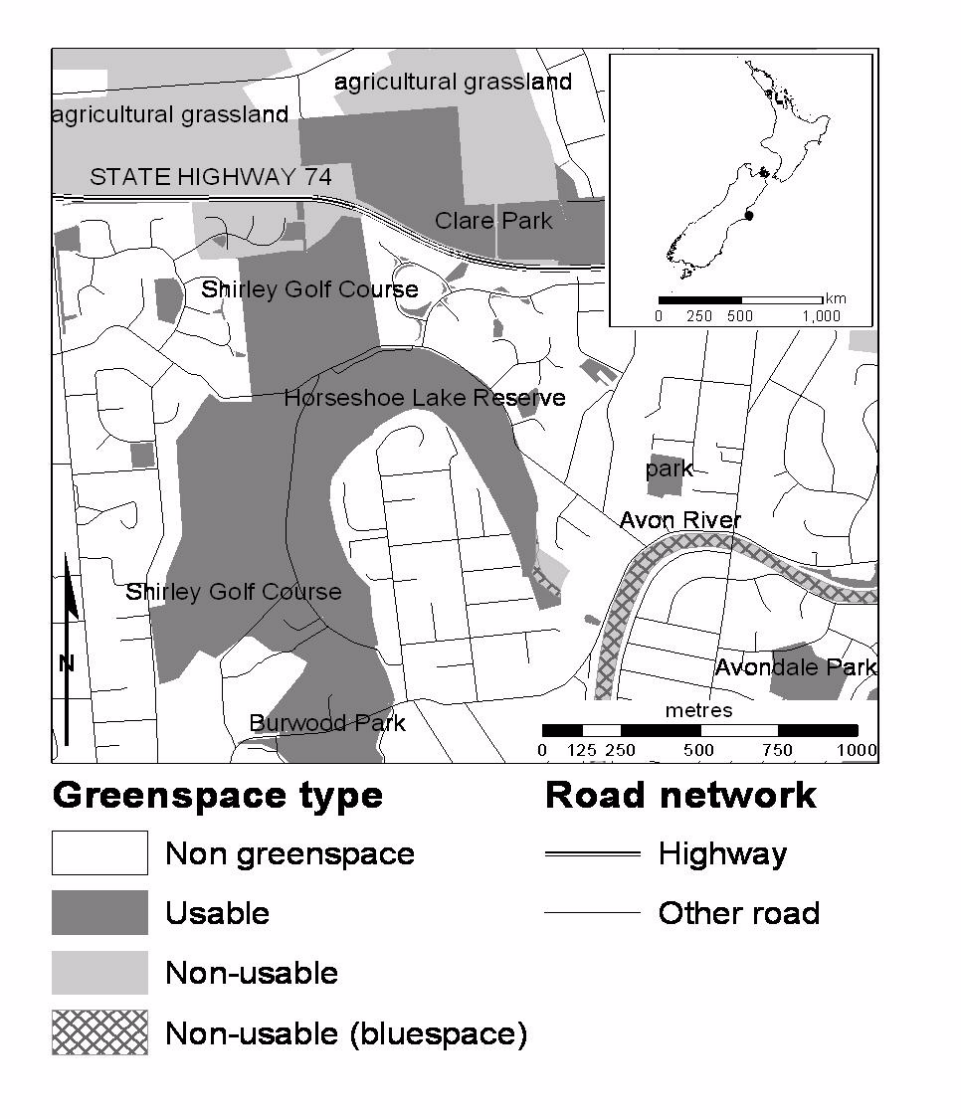
**Extract of the green space classification**. An example of the green space classification for an area in the north east of Christchurch, New Zealand (approximate location indicated by dot on inset map). Map annotation gives the attribute information available for each area, showing that some are identifiable by name (e.g., Burwood Park) while others are identifiable only by the type of land use (e.g. 'park').

An example of the classification is shown in Figure 2. The classification included green spaces ranging in size from large parks to the numerous small 'Recreation Reserves', some at less than 0.02 ha (200 m^2^). These small areas, found largely in built-up areas, were designated by the DOC for local recreation and sporting activities. CAUs in the main urban areas had a mean of 42% total green space coverage (range 0 to 100%), and 17% usable green space coverage (range 0 to 79%).

### Socioeconomic gradient

**Figure 3:**
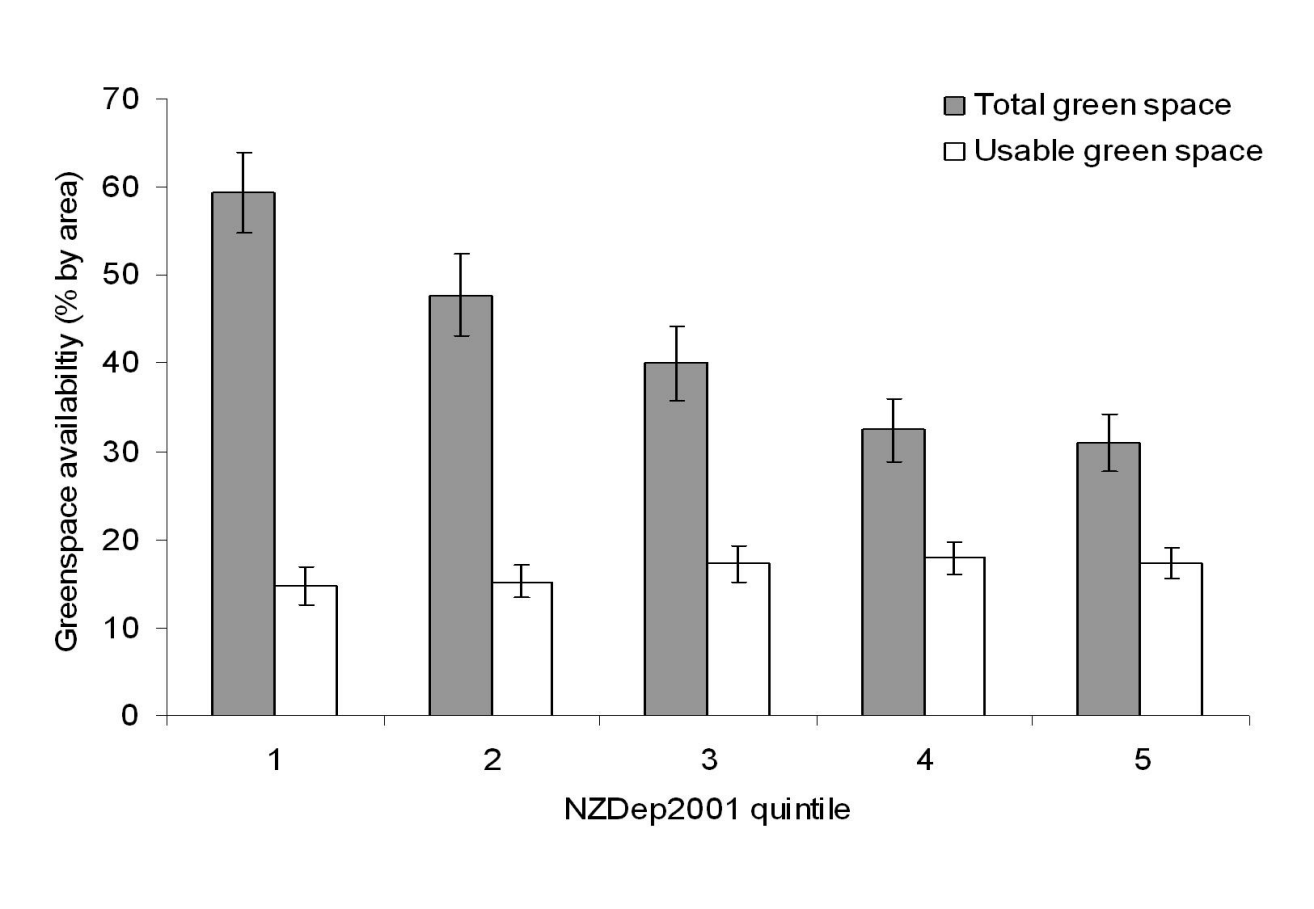
**Green space availability by socioeconomic deprivation**. Mean green space availability by level of socioeconomic deprivation (NZDep2001 quintile). Bars indicate 95% CIs around the mean.

Socioeconomic gradients in green space availability were observed (Figure 3). Figure 3 shows a clear and marked association such that mean total green space availability fell with increasing socioeconomic deprivation. The NZDep2001 score was a significant predictor of percent total green space (ordinary least squares (OLS) regression coefficient = -0.11; *p *< 0.001). In other words, a one standard deviation increase in deprivation score was associated with 11% less total green space. However, the association between deprivation and usable green space was in the opposite direction; greater deprivation was associated with a greater quantity of usable green space (OLS coefficient = 0.02; *p *= 0.003).

### Associations with mortality

Results of the investigation into the relationship between green space and mortality in New Zealand are presented in Tables 2 and 3. Population density quintiles were not significant predictors in any green space and mortality relationships, and did not substantively affect the results (model 5), hence these results are not presented.

**Table 2:** Incidence rate ratios (95% confidence intervals) for cardiovascular disease mortality predicted from (a) total and (b) usable green space availability.

	(a) Total green space			
	**Model 1** **(Baseline)**	**Model 2** **(+ area deprivation)**	**Model 3** **(+ smoking rate)**	**Model 4** **(+ air pollution)**

Green space availability quartile				

1 (least)	1.00	1.00	1.00	1.00

2	1.04 (0.97 to 1.12)	1.03 (0.97 to 1.09)	1.02 (0.96 to 1.08)	1.02 (0.96 to 1.08)

3	1.00 (0.93 to 1.08)	1.06 (1.00 to 1.13)	1.03 (0.97 to 1.09)	1.01 (0.94 to 1.07)

4 (most)	0.86 (0.79 to 0.94)	1.16 (1.07 to 1.25)	1.07 (0.99 to 1.16)	1.01 (0.91 to 1.11)

Sex				

Male	1.00	1.00	1.00	1.00

Female	0.41 (0.39 to 0.43)	0.40 (0.39 to 0.42)	0.40 (0.39 to 0.42)	0.40 (0.39 to 0.42)

Age group				

55 to 64	1.00	1.00	1.00	1.00

45 to 54	0.36 (0.33 to 0.38)	0.35 (0.34 to 0.37)	0.35 (0.34 to 0.37)	0.35 (0.34 to 0.37)

15 to 44	0.06 (0.06 to 0.07)	0.06 (0.06 to 0.06)	0.06 (0.06 to 0.06)	0.06 (0.06 to 0.06)

Area deprivation (NZDep2001)				

1 (least)		1.00	1.00	1.00

2		1.45 (1.33 to 1.59)	1.31 (1.19 to 1.44)	1.31 (1.20 to 1.44)

3		1.89 (1.74 to 2.06)	1.51 (1.36 to 1.68)	1.52 (1.37 to 1.69)

4		2.45 (2.26 to 2.66)	1.77 (1.58 to 1.99)	1.78 (1.59 to 2.00)

5 (most)		3.83 (3.53 to 4.15)	2.48 (2.20 to 2.80)	2.48 (2.20 to 2.81)

Smoking rate				

1 (least)			1.00	1.00

2			1.23 (1.13 to 1.34)	1.23 (1.14 to 1.34)

3			1.35 (1.22 to 1.48)	1.35 (1.22 to 1.48)

4 (most)			1.68 (1.51 to 1.87)	1.66 (1.49 to 1.85)

Air pollution (PM10)				

1 (least)				1.00

2				0.97 (0.89 to 1.06)

3				0.89 (0.81 to 0.98)

4				0.92 (0.83 to 1.01)

5 (most)				0.92 (0.84 to 1.01)

	**(b) Usable green space**			

Green space availability quartile				

1 (least)	1.00	1.00	1.00	1.00

2	1.03 (0.95 to 1.12)	0.95 (0.89 to 1.02)	0.96 (0.90 to 1.03)	0.97 (0.91 to 1.04)

3	1.09 (1.01 to 1.18)	0.95 (0.89 to 1.02)	0.97 (0.90 to 1.03)	0.97 (0.91 to 1.04)

4 (most)	1.07 (0.99 to 1.16)	0.94 (0.88 to 1.01)	0.96 (0.90 to 1.03)	0.96 (0.90 to 1.03)

Sex				

Male	1.00	1.00	1.00	1.00

Female	0.41 (0.39 to 0.44)	0.40 (0.38 to 0.42)	0.40 (0.39 to 0.42)	0.40 (0.39 to 0.42)

Age group				

55 to 64	1.00	1.00	1.00	1.00

45 to 54	0.36 (0.33 to 0.38)	0.35 (0.34 to 0.37)	0.35 (0.34 to 0.37)	0.35 (0.34 to 0.37)

15 to 44	0.06 (0.06 to 0.07)	0.06 (0.05 to 0.06)	0.06 (0.06 to 0.06)	0.06 (0.06 to 0.06)

Area deprivation (NZDep2001)				

1 (least)		1.00	1.00	1.00

2		1.43 (1.31 to 1.56)	1.30 (1.18 to 1.42)	1.31 (1.20 to 1.44)

3		1.86 (1.72 to 2.02)	1.49 (1.35 to 1.65)	1.53 (1.38 to 1.69)

4		2.39 (2.21 to 2.59)	1.74 (1.56 to 1.94)	1.79 (1.60 to 2.00)

5 (most)		3.72 (3.44 to 4.03)	2.42 (2.16 to 2.72)	2.49 (2.22 to 2.81)

Smoking rate				

1 (least)			1.00	1.00

2			1.24 (1.14 to 1.35)	1.24 (1.14 to 1.34)

3			1.37 (1.24 to 1.51)	1.35 (1.22 to 1.48)

4 (most)			1.71 (1.54 to 1.90)	1.66 (1.49 to 1.85)

Air pollution (PM10)				

1 (least)				1.00

2				0.98 (0.90 to 1.06)

3				0.90 (0.83 to 0.97)

4				0.92 (0.85 to 1.00)

5 (most)				0.92 (0.85 to 1.00)

All models adjusted for sex and age-group. Area-level confounders added sequentially in models 2, 3 and 4.

**Table 3:** Incidence rate ratios (95% confidence intervals) for lung cancer mortality predicted from (a) total and (b) usable green space availability.

	(a) Total green space			
	**Model 1** **(Baseline)**	**Model 2** **(+ area deprivation)**	**Model 3** **(+ smoking rate)**	**Model 4** **(+ air pollution)**

Green space availability quartile				

1 (least)	1.00	1.00	1.00	1.00

2	1.11 (0.99 to 1.24)	1.08 (0.98 to 1.20)	1.07 (0.97 to 1.19)	1.11 (0.99 to 1.23)

3	1.02 (0.90 to 1.14)	1.09 (0.98 to 1.22)	1.05 (0.94 to 1.17)	1.09 (0.97 to 1.22)

4 (most)	0.91 (0.79 to 1.05)	1.23 (1.07 to 1.41)	1.10 (0.95 to 1.26)	1.12 (0.94 to 1.32)

Sex				

Male	1.00	1.00	1.00	1.00

Female	0.79 (0.72 to 0.86)	0.78 (0.72 to 0.84)	0.77 (0.71 to 0.84)	0.77 (0.71 to 0.84)

Age group				

55 to 64	1.00	1.00	1.00	1.00

45 to 54	0.27 (0.25 to 0.30)	0.27 (0.25 to 0.30)	0.27 (0.25 to 0.30)	0.27 (0.25 to 0.30)

15 to 44	0.02 (0.02 to 0.02)	0.02 (0.02 to 0.02)	0.02 (0.02 to 0.02)	0.02 (0.02 to 0.02)

Area deprivation (NZDep2001)				

1 (least)		1.00	1.00	1.00

2		1.32 (1.13 to 1.54)	1.13 (0.96 to 1.33)	1.12 (0.94 to 1.32)

3		1.98 (1.71 to 2.29)	1.39 (1.15 to 1.67)	1.38 (1.14 to 1.66)

4		2.52 (2.19 to 2.91)	1.52 (1.24 to 1.87)	1.51 (1.23 to 1.85)

5 (most)		3.64 (3.16 to 4.19)	1.93 (1.56 to 2.40)	2.00 (1.60 to 2.48)

Smoking rate				

1 (least)			1.00	1.00

2			1.33 (1.14 to 1.55)	1.32 (1.13 to 1.53)

3			1.64 (1.37 to 1.96)	1.62 (1.36 to 1.94)

4 (most)			2.09 (1.72 to 2.54)	2.02 (1.66 to 2.46)

Air pollution (PM10)				

1 (least)				1.00

2				0.89 (0.76 to 1.03)

3				0.87 (0.75 to 1.02)

4				0.99 (0.84 to 1.16)

5 (most)				1.04 (0.88 to 1.23)

	**(b) Usable green space**			

Green space availability quartile				

1 (least)	1.00	1.00	1.00	1.00

2	1.06 (0.93 to 1.20)	0.95 (0.84 to 1.08)	0.98 (0.86 to 1.10)	0.99 (0.88 to 1.12)

3	1.11 (0.97 to 1.26)	0.97 (0.86 to 1.09)	0.98 (0.87 to 1.10)	1.00 (0.89 to 1.13)

4 (most)	1.10 (0.97 to 1.25)	0.97 (0.85 to 1.09)	0.99 (0.88 to 1.11)	1.02 (0.90 to 1.15)

Sex				

Male	1.00	1.00	1.00	1.00

Female	0.79 (0.72 to 0.86)	0.77 (0.71 to 0.84)	0.77 (0.71 to 0.84)	0.77 (0.71 to 0.84)

Age group				

55 to 64	1.00	1.00	1.00	1.00

45 to 54	0.27 (0.25 to 0.30)	0.27 (0.25 to 0.30)	0.27 (0.25 to 0.30)	0.27 (0.25 to 0.30)

15 to 44	0.02 (0.02 to 0.02)	0.02 (0.02 to 0.02)	0.02 (0.02 to 0.02)	0.02 (0.02 to 0.02)

Area deprivation (NZDep2001)				

1 (least)		1.00	1.00	1.00

2		1.30 (1.11 to 1.51)	1.12 (0.95 to 1.32)	1.11 (0.94 to 1.31)

3		1.94 (1.68 to 2.24)	1.36 (1.13 to 1.63)	1.36 (1.13 to 1.64)

4		2.45 (2.12 to 2.82)	1.49 (1.22 to 1.81)	1.48 (1.21 to 1.81)

5 (most)		3.51 (3.06 to 4.03)	1.88 (1.53 to 2.31)	1.94 (1.57 to 2.40)

Smoking rate				

1 (least)			1.00	1.00

2			1.35 (1.16 to 1.57)	1.33 (1.14 to 1.55)

3			1.67 (1.40 to 1.99)	1.65 (1.39 to 1.97)

4 (most)			2.14 (1.76 to 2.59)	2.06 (1.70 to 2.50)

Air pollution (PM10)				

1 (least)				1.00

2				0.87 (0.76 to 1.00)

3				0.85 (0.74 to 0.98)

4				0.95 (0.83 to 1.09)

5 (most)				0.99 (0.86 to 1.13)

All models adjusted for sex and age-group. Area-level confounders added sequentially in models 2, 3 and 4.

After controlling for all available confounders we found no relationship between availability of total green space and CVD mortality (Table 2a, model 4). For usable green space availability, all CVD mortality IRRs were lower than 1.0 after accounting for deprivation (models 2 to 4, Table 2b), suggesting mortality rates that were slightly reduced, although not significantly so. Thus, in our study we found no evidence that CVD mortality was related to availability of either total or usable green space in New Zealand CAUs.

Elevated IRRs were found for the relationship between total green space and lung cancer mortality (Table 3a), but wide confidence intervals rendered the findings non-significant. For usable green space, no significant relationship with lung cancer mortality was found, and the IRRs were inconsistent in direction (Table 3b).

## Discussion

This New Zealand study examined the association between green space and mortality using ecological analytical methods. It is the first study to aim to explore the relative importance of causative mechanisms through contrasting relationships between green space and mortality for differing types of green space. We successfully aggregated three data sets to produce a high resolution classification that distinguished usable from non-usable green space. Our classification is the first comprehensive model for New Zealand that differentiates between functional types of green space. Compared with other available national classifications (e.g., the LCDB2) our classification permits the identification of smaller areas of green space that may have local importance and health-relevance.

An important finding from this research was that opposing socioeconomic gradients were observed for the availability of total and usable green space: deprived neighbourhoods were relatively disadvantaged in total green space availability, but had relatively more usable green space. Total green space availability increased markedly with socioeconomic affluence, presumably because the larger, less densely populated and hence greener CAUs on the urban periphery tend to be more affluent. Much of this green space will be agricultural, and therefore classified as non-usable. In contrast, CAUs in densely populated inner-city areas typically have less undeveloped land, but most if not all of the available green space will be usable, hence the reverse socioeconomic gradient we observed for usable green space. This finding concurs with other work that found deprived communities in New Zealand have better geographical access to parks than more affluent areas [29,30].

Our study found no evidence that either total or usable green space availability was related to either cardiovascular disease or lung cancer mortality. The single other known study of green space and mortality found similarly that lung cancer mortality was not associated with green space exposure, but that cardiovascular disease mortality was significantly reduced in greener areas [10]. Additionally, studies that have included related morbidity outcomes have reported protective associations of green space with blood pressure [7], obesity and overweight [8], and coronary heart disease [9]. However, other work from New Zealand has found no relationship between green space and BMI [12], which, in conjunction with our work, may indicate that green space and health relationships in New Zealand differ from those found in other countries.

There are a number of possible explanations for why New Zealand findings might differ. Firstly, there may be a lack of variation in exposure to green space in New Zealand, compared with other countries studied. Average total green space for New Zealand's 'main urban area' CAUs (42%) ranks them similar to the 'slightly urban' areas of Maas et al. [35], indicating that urban areas of New Zealand are greener than those in the Netherlands. Secondly, public green spaces may be less important for health in New Zealand because private gardens tend to be larger, at least when compared with the UK [36,37]. Private gardens were not included in our green space measures because none of the three land cover data sets we used had included them (only large gardens of at least 1 ha would be identified in the LCDB2 data set). Thirdly, aquatic areas ('blue space') may have greater importance for health in New Zealand than elsewhere, as a high proportion of the population (65%) lives within 5 km of the sea [38]. A measure combining green and blue space may therefore be more closely associated with better health than green space alone.

Finally, green space quality may be a better predictor of health than quantity [3,4]. For example, Annear et al. [39] found that residents of an area perceived to have a poor quality physical and social environment engaged in leisure time physical activity less frequently than those living in a higher quality area of the same city. Our measure of green space availability was an objective area-based measure, whereas attributes such as aesthetic quality and perceived safety may also influence the relationship between green space and health [8]. Measuring these qualities would not be possible for a national scale classification such as ours, but their importance should be investigated further, in localised studies. Regardless of their availability to residents, lower quality areas of green space may be less conducive to facilitating physical activity or a restorative experience [3,4].

Our third objective was to investigate the mechanism by which green space may influence mortality outcomes, by contrasting mortality associations for usable and total green space. However, we found no evidence that either type of green space influenced mortality outcomes in New Zealand, hence cannot make inference as to the likely mechanism. Repeating the analyses for contexts in which health associations have been found, and for which usable and non-usable green space types can be differentiated, would provide a useful insight into the mechanism behind the relationship.

Our study had limitations. First, to produce a national classification as objectively as possible we automated the process. Misclassifications were identified using local knowledge and addressed in the automation process, but given the national-level coverage of the dataset it was not possible to correct all minor inconsistencies. Private gardens were necessarily omitted, as discussed above.

Second, the number of non-significant results in the expected direction, for cardiovascular disease in particular, suggested that the models may have lacked the statistical power to detect subtle trends. Residual confounding by unmeasured risk factors that are likely to have a substantial influence on cardiovascular disease (e.g., diet, BMI, alcohol consumption) may have larger influences on the risk of CVD mortality than exposure to green space. Detection of a small effect is difficult, however we did deploy the largest data set available for the investigation of this topic.

Third, we investigated available green space within each CAU but did not consider the health relevance of green space across a wider area to account for travel to green space areas (e.g., using a buffer around each CAU). However, in an ecological study such as this, with no means of quantifying individual exposure to green space outside of the CAU of residence, any attempt to include green space across a wider area would have been subject to similar exposure misclassification issues. As such, the measure of green space we used (% coverage per CAU) captured the green spaces that most residents were likely to experience most often, but cannot be considered a comprehensive measure of green space exposure.

Finally, the distinction between usable and non-usable green space in our classification was relatively coarse, whereas finer level green space type differences may have relevance for health. For example, a large regional park may be used by people from a wide catchment area, but used infrequently, whereas a small local park may serve a smaller catchment area but be used more frequently. Such distinctions could not be made reliably in our classification, but future work could usefully explore the health implications of different green space types.

## Conclusion

We developed a novel classification of green space types, based on the utility of each space (usable or non-usable), and found different socioeconomic gradients in exposure to usable and total green space. We found that public green space availability in New Zealand may not be as important a determinant of health as found elsewhere. Importantly these findings emphasise that green space and health relationships are likely to vary on a nation-by-nation basis. Further investigation of the national variations that contribute to the differences will help inform the wider green space and health debate.

## Competing interests

The authors declare that they have no competing interests.

## Authors' contributions

RM, JP and SK conceived the study. PD and ER acquired and processed datasets. ER conducted the analyses and drafted the manuscript. All authors participated in design and coordination of the study, and all read and approved the final manuscript.

## Pre-publication history

The pre-publication history for this paper can be accessed here:

http://www.biomedcentral.com/1471-2458/10/240/prepub
